# Short and Long Term Outcomes Associated with Fetal Cholelithiasis: A Report of Two Cases with Antenatal Diagnosis and Postnatal Follow-Up

**DOI:** 10.1155/2014/714271

**Published:** 2014-09-30

**Authors:** Juan Troyano-Luque, Ana Padilla-Pérez, Ingrid Martínez-Wallin, Margarita Álvarez de la Rosa, Salvatore Andrea Mastrolia, José Luis Trujillo, Tirso Pérez-Medina

**Affiliations:** ^1^Department of Obstetrics and Gynecology, Ultrasound and Fetal Medicine Unit, University Hospital of Canary Islands, Ctra. Ofra, s/n, Santa Cruz de Tenerife, 38320 San Cristóbal de La Laguna, Spain; ^2^Department of Obstetrics and Gynecology, School of Medicine, University Hospital Policlinico of Bari and University of Bari “Aldo Moro”, Piazza Giulio Cesare 11, 70124 Bari, Italy; ^3^Department of Obstetrics and Gynecology, University Hospital Puerta de Hierro, Calle Manuel de Falla, 1 Majadahonda, 28222 Madrid, Spain

## Abstract

The aims of this study were to present and discuss ultrasound findings of prenatal fetal cholelithiasis in two cases with different etiology and evolution. Case 1: a pregnant woman from sub-Saharan Africa, suffering from Lyme disease, was treated with ceftriaxone sodium. Six weeks later, biliary sludge associated with polyhydramnios was detected in the fetus and the fetal growth percentile was 14. Emergency caesarean was performed at 36 weeks of gestation due to fetal distress. Biliary sludge persists in the two-and-a-half-year-old child. Case 2: the fetus of a Caucasian woman with normal pregnancy showed multiple cholelithiasis associated with polyhydramnios at 31 weeks of gestation. At 39 weeks and 4 days, cesarean section was performed due to lack of dilation. The biliary disease resolved spontaneously at seven months of age, with no associated abnormalities. In conclusion, prenatal diagnosis of cholelithiasis is straightforward, but prognosis cannot be defined yet. Serious complications do not arise in 70% of cases, but severe diseases may ensue in 20%. Persistence of cholelithiasis after one year of age results in cholelithiasis in childhood and beyond. Biliary sludge is associated with worse prognosis than cholelithiasis when it appears before 28 weeks of gestation.

## 1. Introduction

Prenatal diagnosis of cholelithiasis can be readily performed with sufficient experience and when the genetic sonogram is used.

The gallbladder should be examined routinely using high-resolution ultrasound. This is done primarily from the second trimester, since gallbladder changes are often prenatally underdiagnosed. Importantly, such changes may be a marker of disease in some severe cases [1–3].

## 2. Case 1

The first case involved a 31-year-old pregnant illegal immigrant from Mali with a history of a caesarean birth due to preeclampsia. At 20 weeks of gestation, she was admitted to our department for fever of 38.8°C of unknown origin. The pathology was initially attributed to a nonspecific viral illness. The genetic sonogram showed a fetus without signs of malformation, except for increased gallbladder volume (appropriate for gestational age) and polyhydramnios. Genetic analysis of the amniotic fluid extracted by amniocentesis showed the resulting karyotype was 46 XX.

The patient's symptoms exacerbated rapidly: her fever increased to 40°C and she presented general malaise, facial paresis, cardiac arrhythmia, respiratory distress, joint pain, and stiff neck. Laboratory tests showed slightly elevated erythrocyte sedimentation rate (ESR) and mild leukocytosis as well as lymphocytic pleocytosis with increased IgG and specific oligoclonal bands. Lumbar puncture was performed as the woman had retronucal pain and stiffness. Lymphocytic pleocytosis was detected in cerebrospinal fluid (CSF) with a high concentration of protein and glucose and specific oligoclonal bands. These results and the geographical origin of the patient suggested severe sepsis. Cell cultures such as BSK-Hrevealed infection by* Borrelia *spp., confirmed by polymerase chain reaction (PCR).

The patient was diagnosed with Lyme disease, stages 2 and 3. Intravenous ceftriaxone sodium was administered at doses of 500 mg/mL every 24 hours for 16 days, which effectively cured the infection.

**Figure 1:**
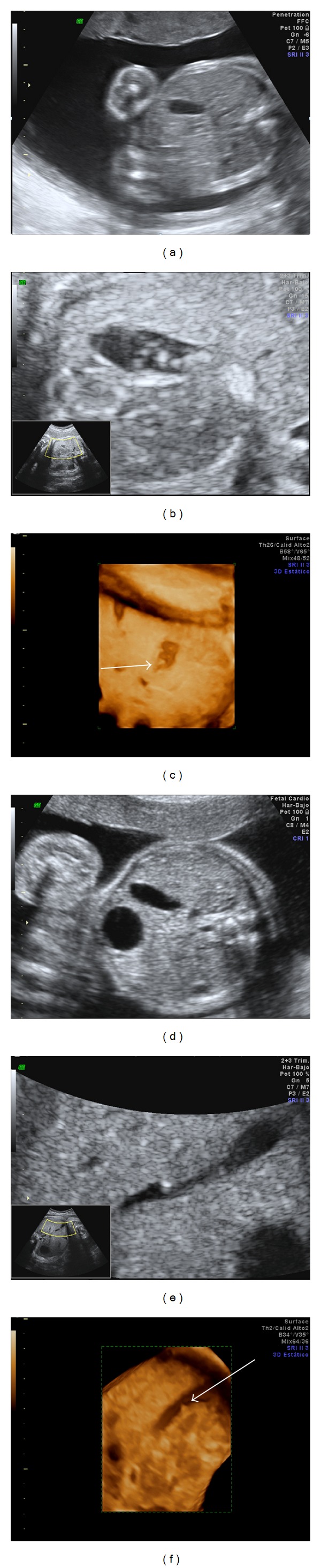
Case 1: ultrasound images of the distended gallbladder (a) containing biliary sludge (b) and 3D ultrasound of the same finding (arrow) (c). Case 2: ultrasound images of the distended gallbladder (d) showing biliary calculi (e) and 3D ultrasound of the same finding (arrow) (f).

At week 26 a distended gallbladder with echogenic content of lumpy consistency was observed (Figure 1(a)). In addition, the fetal growth percentile decreased to percentile 14.

Gestation continued with no significant events but fetal growth almost reached a plateau and gallbladder findings persisted. At week 36, urgent caesarean was required due to fetal distress after fetal growth stopped and pathological hemodynamic data were identified. The female newborn weighed 2090 g and had an Apgar score of 9/9 and umbilical cord pH was 7.25.

In the first hours after birth, the newborn experienced bilious vomiting, so, in addition to being breastfed, she received neonatal serotherapy for three days. The infant showed gradually increasing tolerance to oral feeding, beginning with artificial milk, although there was some sporadic vomiting due to reflux. Positive health developments and a ponderal growth in the 25th percentile allowed hospital discharge at 25 days of birth with a weight of 2,450 grams.

Currently, at two years and six months of age, the infant still suffers from cholelithiasis and has suffered several episodes of typical food intolerance. During this time she has been admitted twice to the hospital emergency department, once for viral meningitis without sequelae and on another occasion for dehydration after a period of acetonemic vomiting. Ponderal index percentile remains low. Cholecystectomy and other possibilities are being considered to treat the infant, depending on short term developments. The infant is currently being studied in the pediatric ward of the hospital.

## 3. Case 2

The second case involved a 31-year-old patient in her second pregnancy, with unremarkable medical history except for one spontaneous miscarriage one year before.

Pregnancy control was performed from 6 weeks of gestation with no significant events and micronized progesterone was vaginally administered at a dose of 200 mg every 24 hours until week 11.

At week 20, moderate polyhydramnios was detected in the fetus and, remarkably, increased gallbladder size was observed, shown in Figure 1(b). Cytogenetic study of amniotic fluid obtained by amniocentesis revealed a 46 XY karyotype. Standard gestational control was performed, focused on regular monitoring of the gallbladder findings. All maternal laboratory results were normal.

At week 32, amniotic fluid volume had normalized but echogenic structures in the distended gallbladder were observed. No pathological intrahepatic signs were detected.

Scheduled ultrasound showed a gradual increase of echogenic structures in the gallbladder. At 39 + 3 weeks of gestation, the patient spontaneously initiated labor which was terminated by cesarean due to fetal distress. The male newborn weighed 3200 g and had an Apgar score of 9/9 and umbilical cord pH was 7.27.

**Figure 2:**
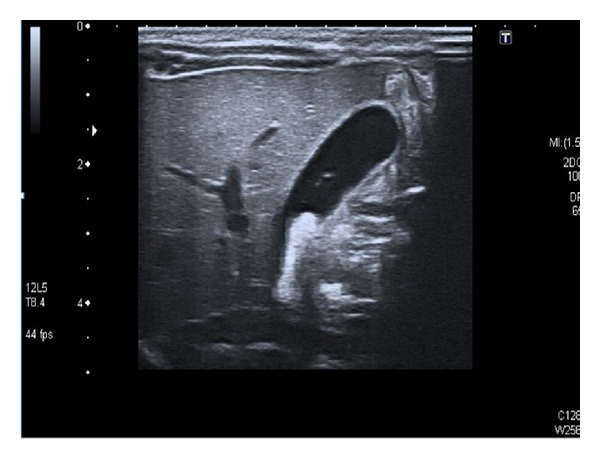
Case 2: neonatal ultrasound image showing fetal gallbladder sludge obstructing the bile duct.

Neonatal ultrasound examination confirmed the presence of cholelithiasis that did not affect the walls of the gallbladder and associated condensed biliary sludge (Figure 2).

Neonatal monitoring showed intolerance to breast milk so feeding with low-fat formula milk was initiated. Breast milk intolerance was associated with low weight gain in the first months, with a ponderal index in the 14th percentile. At 7 months, the cholelithiasis disappeared together with all condensed bile, and the gallbladder presented clear walls.

Currently, the child of 2 years and 6 months is completely asymptomatic and has adequate food tolerance without associated disorders.

## 4. Discussion

The first ultrasound report of prenatal cholelithiasis dates from 1983 [4], but it was Potter in 1928 [5] who first published the presence of fetal cholelithiasis in a neonatal autopsy. Over the past 18 years, we have detected 16 cases of prenatal echogenic material in the gallbladder. In 10 cases where cholelithiasis was detected after 30 weeks of gestation, it resolved in the first year of life without subsequent complications. In two cases it was necessary to perform cholecystectomy at 11 and 14 years of age, respectively, due to acute cholecystitis. The remaining four cases presented complete atrioventricular canal, polycystic kidney disease with corticomedullary dysplasia, primary hyperoxaluria (neonatal diagnosis), and genodermatosis (neonatal diagnosis). In these cases, the diagnoses were made by ultrasound before week 24 and neonatal exitus occurred.

Based on the ultrasound diagnosis, cholelithiasis can occur at any time during pregnancy, but early gestational age and maternal or underlying family-specific diseases must be considered when establishing criteria for prenatal cholelithiasis severity. On the one hand, the appearance of biliary sludge before 26 weeks was a factor of neonatal severity and chronicity in one of the cases. On the other hand, the appearance of cholelithiasis after 30 weeks of gestation, without any risk factors, was associated with good prenatal and neonatal clinical course.

Genetic disorders, racial and environmental variables, socioeconomic status, poor nutrition, maternal dehydration, and sepsis should be considered as risk factors affecting the severity of fetal cholelithiasis [6].

About 70% of cholelithiasis cases are diagnosed postnatally [7]; fortunately most are not significantly linked to severe perinatal conditions [1–3], but based on our experience at least the relationship of cholelithiasis with child choledocholithiasis should be considered, as noted by other authors [8].

Remarkably, in our two cases, increased gallbladder volume and polyhydramnios appeared before the echogenic material inside was detected; therefore we could consider these as signs appearingbefore the onset of cholelithiasis.

From a clinical point of view, a better outcome was observed in the male fetus (Case 2) with later onset of cholelithiasis and whose mother had no significant medical history.

The only repeated event of some significance in both pregnancies of the sub-Saharanpatient, noting the previous miscarriage, was the transvaginal micronized progesterone administered from the 6th to the 11th week of gestation.

In the first case the mother had multiple risk factors, including the absence of obstetric control until week 20 of pregnancy, being an illegal immigrant from sub-Saharan Africa, and presenting fever upon hospital admission with subsequent worsening of her general health. At the beginning, the pathogen responsible for the deterioration of her general condition could not be identified by standard cell culture techniques. However, one of the special culture media used, BSK-H, suggested it could be a* Borrelia *spp. infection.

PCR and reverse line blot hybridization assay were used to diagnose Lyme disease in this patient. Lyme disease is caused by the bacterium* Borrelia burgdorferi* and transferred to humans through a tick vector such as blacklegged ticks that have previously bitten rodent carriers. It was first detected in Old Lyme in CT, USA, hence its name [7]. The disease has three evolutionary stages: stage 1, early localized Lyme disease; stage 2, early disseminated infection; and stage 3, late persistent Lyme disease. The latter is regarded as the acute phase of the disease. The factors associated with increased likelihood of infection are living outdoors, in rural settings, and in close contact with domestic animals or pets (such as primates), essentially in those places where it is possible to be bitten by a tick of this type. Poor hygiene or low socioeconomic status increases the probability of Lyme disease infection.

A striking finding in the first case was the presence of dense biliary sludge in the 26th week, undetected in previous examinations. Besides the described infection, the only probable causal factor of the fetal disease was the administration of ceftriaxone sodium for 14 days at doses of 500 mg/mL every 24 hours. This drug belongs to the group of cephalosporins and there are clear contraindications to its use in cases of jaundice, acidosis, or hypoalbuminemia: under these conditions the drug prevents bilirubin from binding to serum albumin. This action is enhanced by drugs that contain calcium. Treatment duration of longer than 14 days, together with renal failure or dehydration, can lead to precipitation of ceftriaxone calcium in the gallbladder. Cholelithiasis usually disappears when treatment is stopped [9]. It is therefore not advisable to administer doses higher than 80 mg/kg, as this increases the risk of biliary precipitation. Pancreatitis is one of the disease complications in these patients [10].

The incidence of prenatal cholelithiasis is one case per 260 [11], but despite technological advances, 70% are not diagnosed. Fortunately this condition is not life-threatening for most children, although it is a cause of choledocholithiasis in childhood and preadolescence [12, 13]. However, 20% of fetal cholelithiasis cases can be associated with primary pulmonary hypertension, renal ectopia, and hydrocephalus [3]. Other authors point out that in 30% of cases cholelithiasis is associated with hydronephrosis and hemivertebra syndrome [3] and even claim that 100% of cholelithiasis cases are associated with major malformations. These data should make us aware that, at least in 20% of cases, prenatal cholelithiasis should be considered as a marker of severe fetal malformation or disease.

Cholelithiasis has not been linked with maternal age, parity, or perinatal pathology in 80% of cases, although 12% of mothers present choledocholithiasis [2]. Incidence of cholelithiasis is three times higher in girls than in boys. Diagnosis is usually made after 30 weeks of gestation in cases of a positive gestation course. The most common neonatal diseases associated with fetal cholelithiasis and some triggers are summarized in Table 1.

Biliary sludge is more likely to persist in neonates in the long term, with an 18% risk, as opposed to cholelithiasis, with a 2% risk [14]. Most cases (87%) spontaneously resolve within the first 3 and 7 months of birth. Most are asymptomatic, but in some cases there can be gastrointestinal symptoms, leading to particular diets to avoid food intolerances and even cholecystectomy in childhood and puberty [15]. Biliary sludge can be a risk factor for acute cholecystitis in childhood and adolescence or hepatobiliary disease in adulthood. For this reason, a conservative prenatal approach and regular neonatal monitoring during the first year of life is recommended.

The appearance of echogenic material in the gallbladder before 26 weeks of gestation may be associated with pancreatic cystic fibrosis, so early onset carries a worse prognosis [16]. Cholelithiasis generally has a better prognosis than biliary sludge. However, if biliary sludge appears before 26–24 weeks of gestation it may be due to Caroli's syndrome, an extremely severe congenital disease [17].

## 5. Conclusion

Although fetal cholelithiasis does not imply a severe disease, 20% of cases can be associated to genetic disorders, malformations, or other diseases.

Biliary sludge is associated with worse prognosis than cholelithiasis, and its early onset may signal the presence of Caroli's disease and epidermal nevus syndromes.

Polyhydramnios and increased gallbladder volume often appear at the beginning of the 2nd trimester, before the appearance of cholelithiasis. Certain drugs including ceftriaxone sodium can induce the appearance of biliary echogenic material.

The association of sepsis and poor maternal dietary intake may be predisposing factors for long term cholelithiasis in children. About 11% of prenatal cholelithiasis cases precede cholelithiasis and cholecystitis in children and adults. Over 70% of cases of cholelithiasis are not detected prenatally, so this disorder should be included in the genetic sonogram assessment.

## Figures and Tables

**Table 1:** Neonatal diseases linked to fetal cholelithiasis.

Hemolytic disease (10%)	
Fetal obesity-macrosomia (1%-2%)	
Pancreatic cystic fibrosis (4%–6%)	
Cholestasis (70%)	
Chronic liver disease	
Down syndrome. Other chromosome disorders (2%)	
Hypercholesterolemia (2%)	
Congenital malabsorption syndrome	
Ceftriaxone treatment (5%-6%)	
Maternal anticancer drug treatment	
Prematurity	
Prolonged maternal fasting and dehydration (Third World countries)	
